# Frequency of TERT Promoter Mutations in Ameloblastoma: A Retrospective Study

**DOI:** 10.3390/diagnostics16071078

**Published:** 2026-04-02

**Authors:** Mee-seon Kim, Shin-Ah Son, So-Young Choi

**Affiliations:** 1 Department of Pathology, School of Dentistry, Kyungpook National University Hospital, Kyungpook National University, Daegu 41944, Republic of Korea; meeseonkim@knu.ac.kr; 2 Trauma Center, Department of Thoracic and Cardiovascular Surgery, School of Medicine, Kyungpook National University Hospital, Kyungpook National University, Daegu 41944, Republic of Korea; 3 Department of Oral and Maxillofacial Surgery, School of Dentistry, Kyungpook National University Dental Hospital, Kyungpook National University, Daegu 41940, Republic of Korea

**Keywords:** ameloblastoma, TERT promoter mutation, TERT, telomerase reverse transcriptase, frequency

## Abstract

Telomerase reverse transcriptase (TERT) plays a key role in tumorigenesis by maintaining telomere length, promoting chromosomal stability, and enabling cells to evade replicative senescence. TERT promoter mutations have been detected in various types of tumor; however, their prevalence in ameloblastoma has not been verified. This study aimed to determine the frequency of TERT promoter mutations in ameloblastoma. This retrospective study included formalin-fixed, paraffin-embedded (FFPE) tissue specimens and corresponding medical records from patients who underwent surgical treatment for jaw ameloblastoma at the Department of Oral and Maxillofacial Surgery, Kyungpook National University (Daegu, Republic of Korea) between January 2011 and December 2024. Clinical data were reviewed through January 2026. Of the 49 patients included, genomic DNA was extracted from two 5 μm thick FFPE tissue sections using the PANAMAX™ FFPE Plus DNA Extraction Kit (HLB PANAGENE, Daejeon, Republic of Korea), according to the manufacturer’s instructions. Hotspot TERT promoter mutations (C228T and C250T) were analyzed using the PNAClamp™ TERT Mutation Detection Kit (HLB PANAGENE, Daejeon, Republic of Korea). From a total of 73 TERT promoter mutation analyses performed in 49 patients, one of the recurrent cases harbored both C228T and C250T hotspot mutations. In the non-recurrent group, one case exhibited a C250T mutation. These findings indicate that TERT promoter mutations are rare in ameloblastoma.

## 1. Introduction

Telomeres are specialized nucleoprotein structures located at the ends of linear chromosomes that safeguard genomic stability and limit cellular replicative capacity [1,2]. In most somatic cells, progressive telomere shortening acts as an intrinsic tumor-suppressive mechanism, as telomerase activity is largely absent in differentiated human cells owing to transcriptional silencing of the telomerase reverse transcriptase (TERT) gene [3]. TERT is a key driver of cancer development, as it maintains telomere length, promotes chromosomal stability, and allows cells to evade replicative senescence. Over 80% of tumors achieve telomere maintenance through various regulatory mechanisms, among which TERT promoter mutations represent one of the most frequent alterations [4]. A previous report indicates that TERT promoter mutations occur at high frequencies (up to 80–90%) in glioblastoma, melanoma, urothelial bladder cancer, myxoid liposarcoma, and medulloblastoma, and at intermediate frequencies (15–50%) in thyroid, hepatocellular, upper tract urothelial, head and neck, and ovarian clear cell carcinomas [3]. In contrast, the prevalence of TERT promoter mutations in ameloblastoma has not been well established, with only a few small-scale studies reported to date [5,6]. Therefore, the present study aimed to determine the frequency of TERT promoter mutations in ameloblastoma and evaluate their clinicopathologic significance.

## 2. Materials and Methods

### 2.1. Patients

This retrospective, single-center study analyzed archived histopathologic specimens from patients diagnosed with ameloblastoma. This retrospective study included formalin-fixed, paraffin-embedded tissue blocks and corresponding medical records from patients who underwent surgical treatment for ameloblastoma at the Department of Oral and Maxillofacial Surgery, Kyungpook National University, between January 2011 and December 2024 (Daegu, Republic of Korea). The study cohort was restricted to central (intraosseous) ameloblastomas arising in the jaw bones, while peripheral (extraosseous) ameloblastomas were excluded from the analysis. Medical records were collected and reviewed from January 2011 through January 2026. Clinical data were obtained from medical records, and corresponding pathology reports were also reviewed to verify diagnoses and collect detailed histopathologic information. The study protocol was approved by the Institutional Review Board of Kyungpook National University Hospital (IRB No. KNUH 2026-01-022; 3 February 2026). In addition, the requirement for written informed consent was waived due to the retrospective nature of this study.

**Figure 1:**
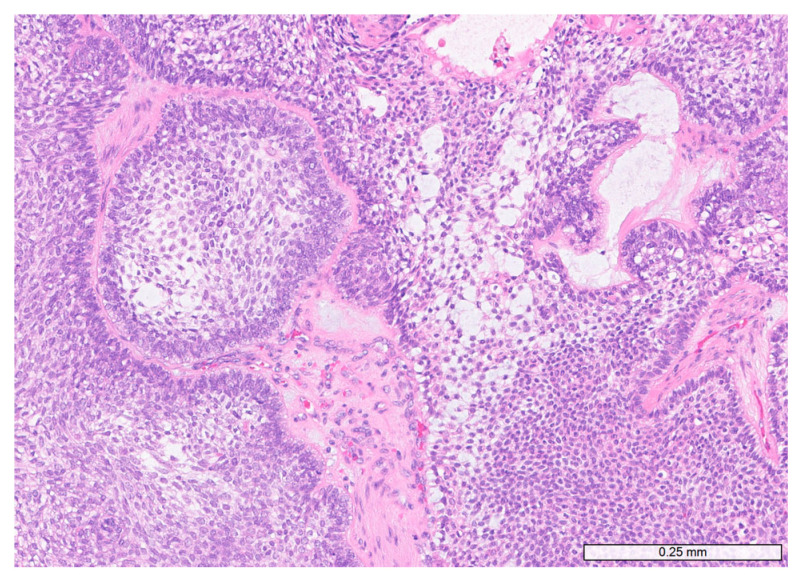
**Representative histopathologic features of ameloblastoma.** Tumor islands composed of peripheral palisading columnar cells with reverse nuclear polarity and central stellate reticulum-like cells (hematoxylin and eosin stain; ×100).

All available histologic slides from 107 patients initially diagnosed with ameloblastoma were independently reviewed by two experienced surgical pathologists (M.S.K. and S.Y.C.) in accordance with the World Health Organization (WHO) Classification of Tumours of the Head and Neck, 5th edition. Diagnostic criteria included odontogenic epithelial proliferation with peripheral palisading of columnar or cuboidal cells exhibiting reverse nuclear polarity and subnuclear vacuolization, as well as a central stellate reticulum-like component. Representative microscopic features of ameloblastoma are shown in Figure 1.

**Figure 2:**
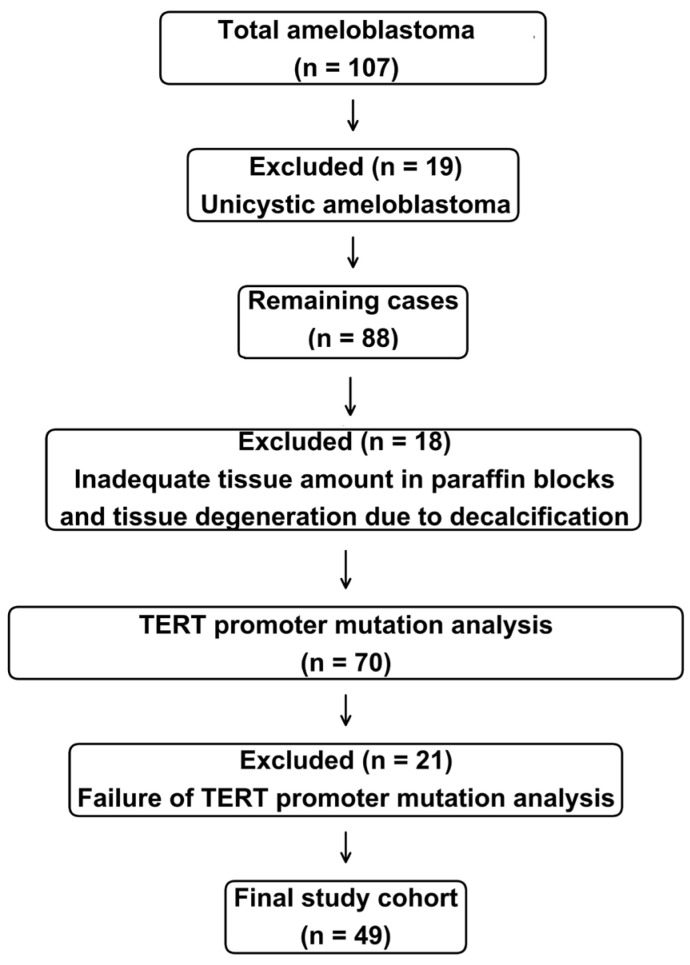
**Study cohort selection.** Histologic slides from 107 patients initially diagnosed with ameloblastoma were independently reviewed according to the WHO classification (5th edition). Nineteen unicystic ameloblastomas were excluded. Of the remaining 88 patients, 18 were further excluded due to insufficient tissue or degradation related to decalcification. TERT promoter mutation analysis was attempted in 70 patients, but failed in 21 patients. A total of 49 patients were finally included in the study.

Following histopathologic review, 19 patients who met the diagnostic criteria for unicystic ameloblastoma were excluded. Among the remaining 88 patients, an additional 18 were excluded due to insufficient tissue in paraffin blocks or significant tissue degeneration caused by decalcification. TERT promoter mutation analysis was subsequently attempted in the remaining 70 eligible patients; however, the analysis failed in 21 patients. After applying all histopathologic and molecular selection criteria, a total of 49 patients were ultimately included in the final study cohort (Figure 2).

**Figure 3:**
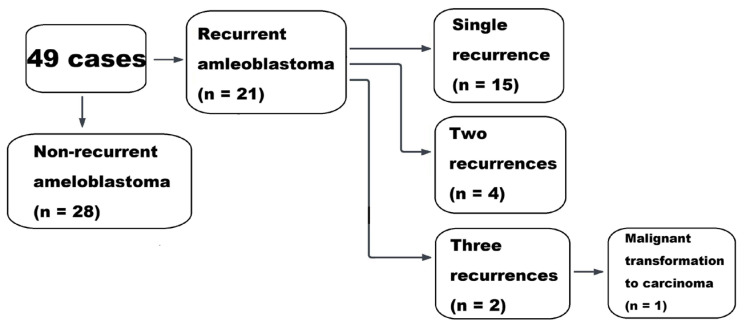
**Flow chart showing recurrence status.** Among the 49 patients included, 21 were recurrent ameloblastomas, including 3 with a prior history before the study period and 18 where recurrence was found during the study period (2011–2026). Frequency of recurrence was single in 15 patients, double in 4 patients, and triple in 2 patients. One patient with multiple recurrences showed malignant transformation to ameloblastic carcinoma at the second recurrence. The remaining 28 patients were newly diagnosed.

Of the 49 patients included in the study, 21 were recurrent ameloblastoma. Among these, 3 patients had a documented history of ameloblastoma at the same jaw location before the study period, while 18 patients developed recurrence during the study period (January 2011 to January 2026). Among the 21 patients who were diagnosed with recurrence, 15 experienced a single recurrence, 4 experienced two recurrences, and 2 experienced three recurrences. One patient with triple recurrence subsequently underwent malignant transformation to ameloblastic carcinoma, which occurred at the second recurrence. The remaining 28 patients were newly diagnosed with ameloblastoma during the study period (Figure 3).

Of the 18 patients who experienced recurrence during the study period, 2 were excluded from the experimental analysis because recurrence occurred in 2025, beyond the study period approved by the Institutional Review Board. In the remaining 16 recurrent cases, TERT promoter mutation analysis was performed on both the primary tumors and the corresponding recurrent lesions. Among the 16 patients diagnosed with recurrence, 10 experienced a single recurrence, 4 experienced two recurrences, and 2 experienced three recurrences, including 1 patient who underwent malignant transformation to ameloblastic carcinoma at the second recurrence. Three patients with a documented history of ameloblastoma at the same site prior to the study period were included, and only the recurrent tumor specimens were available for analysis. In addition, for two patients who recurred in 2025, only the primary tumor specimens were analyzed, as recurrent tumor tissues were unavailable. When combined with the 28 patients who did not experience recurrence, a total of 73 TERT promoter mutation analyses were conducted.

### 2.2. TERT Promoter Mutation Analyses

Genomic DNA was extracted from formalin-fixed, paraffin-embedded (FFPE) tissue blocks using two 5 μm thick sections and the PANAMAX™ FFPE Plus DNA Extraction Kit (HLB PANAGENE, Daejeon, Republic of Korea) according to the manufacturer’s instructions. TERT promoter mutations (C250T and C228T) were analyzed using the PNAClamp™ TERT Mutation Detection Kit (HLB PANAGENE, Daejeon, Republic of Korea) following the manufacturer’s protocol.

Real-time PCR was performed using the CFX96 Real-Time PCR Detection System (Bio-Rad, Hercules, CA, USA), with an input of 10 ng DNA and a SYBR Green-based master mix containing mutation-specific primers and probes, following the manufacturer’s protocol. Cycling conditions were applied according to standard recommendations. The assay has an analytical sensitivity (limit of detection) of approximately 5% mutant allele frequency (MAF), as specified by the manufacturer. The peptide nucleic acid (PNA) probe was designed to selectively bind to the wild-type sequences of TERT C250T and C228T, thereby suppressing the amplification of wild-type alleles. In the presence of mutant alleles, mismatches between the PNA probe and the target DNA sequence prevented suppression, allowing preferential amplification of mutant DNA. SYBR Green fluorescence was measured at each extension step, and threshold cycle (Ct) values were determined automatically. Mutation status was interpreted using the ΔCt value, defined as the difference between the standard Ct and the sample Ct. A ΔCt value > 2.0 was considered indicative of a TERT promoter mutation.

### 2.3. Statistical Analysis

All statistical analyses were performed using R software (version 4.3.1; Posit Software, PBC, Boston, MA, USA). The Hmisc and survival packages were used for statistical analyses. Differences in clinical characteristics between recurrent and non-recurrent cases were evaluated using Fisher’s exact test for categorical variables and the Wilcoxon rank-sum test for continuous variables. A *p*-value < 0.05 was considered statistically significant.

## 3. Results

### 3.1. Clinical Characteristics of Patients

Clinicopathologic data from 46 patients were collected from patient medical records, excluding three cases with a prior history of ameloblastoma before the study period, as information on the primary tumor was unavailable. The patients’ ages ranged from 8 to 79 years (median, 27 years). The median follow-up duration was 2422 days (range, 1041–5182 days), and tumor size ranged from 0.9 to 9.6 cm (median, 3.4 cm). Of the 46 cases, 39 (84.8%) arose in the mandible, and 7 (15.2%) occurred in the maxilla. Among the 18 patients who experienced recurrence, recurrence-free survival ranged from 52 to 3591 days (median, 889 days).

Recurrence was significantly associated with sex. Male patients showed a higher recurrence rate compared with female patients (61% vs. 25%, *p* = 0.03). The median age was higher in the recurrence group than in the non-recurrence group (46.5 years [range, 10–79] vs. 23 years [range, 8–77]); however, this difference was not statistically significant (*p* = 0.2). Regarding tumor location, maxillary tumors demonstrated a higher recurrence rate than mandibular tumors (71% vs. 33%), although the difference did not reach statistical significance (*p* = 0.09). The median tumor size was 2.5 cm (range, 1.0–7.0) in the recurrence group and 3.5 cm (range, 0.9–9.6) in the non-recurrence group, with no significant difference between the groups (*p* = 0.3) (Table 1).

### 3.2. TERT Promoter Mutation Status in Ameloblastoma

**Figure 4:**
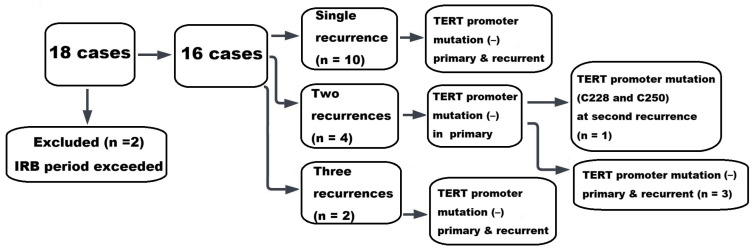
Results of TERT promoter mutation analysis in cases of recurrence.

**Figure 5:**
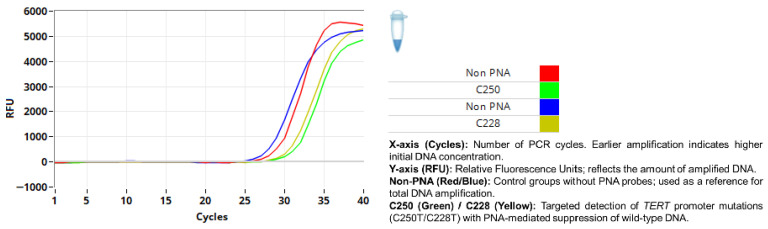
Detection of C228T and C250T mutations.

**Figure 6:**
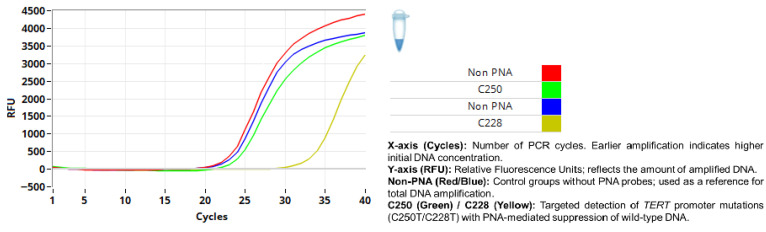
Detection of C250T mutation.

TERT promoter mutations were identified in 2 out of 73 analyses. A compact summary of TERT promoter mutation status across episodes of disease is presented in Table 2 for TERT promoter mutation cases. Among a total of 73 TERT promoter mutation analyses performed in 49 patients, 1 of the 16 patients who experienced recurrence harbored both C228T and C250T hotspot mutations (Figure 4). In the single-recurrence group, no TERT promoter mutation was detected in either the primary or recurrent tumors. Among the four patients with two recurrences, the TERT promoter mutation was not identified in the primary tumor, although one patient harbored C228 and C250 mutations at the second recurrence (Figure 5). The remaining three patients showed no evidence of TERT promoter mutations in primary or recurrent tumors. No TERT promoter mutations were detected in either the primary or recurrent lesions in the two patients with three recurrences, including one case with malignant transformation at the second recurrence. In the non-recurrent group (28 patients), one patient had a C250T mutation (Figure 6). The three patients in whom only recurrent lesions were analyzed (due to a history of ameloblastoma prior to the study period) were all found to be negative for TERT promoter mutations. In addition, two patients in whom only the primary tumors were analyzed (due to recurrence occurring in 2025) were also negative for TERT mutations.

## 4. Discussion

TERT activation plays a central role in cellular immortalization and malignant transformation by maintaining telomere length and overcoming replicative senescence; accordingly, TERT expression and/or telomerase activity is detectable in up to 90% of human primary cancers [3]. Ameloblastoma is a benign odontogenic neoplasm with locally aggressive behavior. Compared with other benign odontogenic tumors, it shows a more infiltrative growth pattern and a higher propensity for recurrence, even after aggressive treatment [7,8]. The BRAF V600E mutation is the most common genetic alteration identified in ameloblastoma [9]. BRAF V600E mutations are frequently detected in ameloblastoma, generally ranging from 60% to 80% [9,10,11,12,13,14]. BRAF mutations are considered a major driver in the pathogenesis of ameloblastoma [15,16]. The coexistence of BRAF V600E and TERT promoter mutations has been frequently reported across multiple cancer types [17,18,19,20]. Several previous studies have elucidated the molecular mechanism linking BRAF V600E mutation to TERT promoter activation [17,20]. Mechanistically, BRAF V600E activates the MAPK/ERK pathway, promoting TERT reactivation by maintaining an open chromatin state at the TERT promoter through GABPA- and Sp1-mediated mechanisms [17,20].

Given the high frequency of BRAF mutations in ameloblastoma [9,10,11,12,13,14], it was hypothesized that TERT promoter mutations might also occur at a meaningful frequency, providing the rationale for this study. However, among a total of 73 cases analyzed, TERT promoter mutations were identified in only two cases, indicating that such alterations are rare in ameloblastoma despite the frequent presence of BRAF mutations.

There have been only a few previous studies examining TERT promoter mutations and telomerase activity in ameloblastoma. A previous study using immunohistochemistry demonstrated that all ameloblastoma samples showed positive telomerase activity [5]. A previous study employed a non-radioactive polymerase chain reaction-based assay to evaluate telomerase activity in various tissue specimens obtained from the oral cavity. Telomerase activity was detected in all four frozen fresh ameloblastoma tissue samples, whereas all three oral rinse specimens were negative for telomerase activity [21]. According to a previous study, genomic DNA extracted from paraffin-embedded ameloblastomas (*n* = 6) and ameloblastic carcinomas (*n* = 3) were analyzed using Sanger sequencing to assess hotspot TERT promoter mutations (C228T and C250T). None of the samples harbored TERT promoter mutations [6]. This most closely resembles the present study in both its methodological approach and results.

Despite the existence of several previous studies, their small sample sizes and inconsistent findings make it difficult to draw definitive conclusions regarding the role of TERT promoter mutations and telomerase activity in ameloblastoma. Based on the present findings and the limited evidence available to date, the frequency of TERT promoter mutations in ameloblastoma appears to be low.

This study has several limitations. First, the retrospective, single-center design may introduce selection bias and limit the generalizability of the findings. Second, the substantial attrition of cases from the initially identified cohort to the final study population raises the possibility of selection bias. This reduction was primarily due to insufficient tissue, DNA degradation—particularly in decalcified FFPE specimens—and failed molecular analyses. These factors may have influenced the representativeness of the final cohort and should be considered when interpreting the results. Although FFPE tissues are commonly used for molecular analysis and are generally adequate for targeted assays, our results indicate that their suitability may be limited by DNA degradation and technical failure, particularly in decalcified samples. Accordingly, FFPE specimens cannot be considered a complete replacement for fresh or frozen tissues, which remain preferable when optimal DNA quality is required. Third, the extremely low frequency of TERT promoter mutations in this study limits the ability to draw meaningful inferential conclusions. Although clinical differences between the recurrent and non-recurrent groups of ameloblastoma were statistically analyzed (Table 1), TERT promoter mutations were identified at a very low frequency. Therefore, no definitive conclusions could be drawn regarding their clinical significance in this study, and the findings should be considered exploratory. Finally, the use of a PCR-based, mutation-specific assay restricts detection to predefined hotspot mutations and does not allow comprehensive mutational profiling or precise quantification of variant allele frequency. These limitations should be accounted for when interpreting the results.

In conclusion, this study demonstrates that TERT promoter mutations occur at a low frequency in ameloblastoma, suggesting a limited role in its tumorigenesis. Further studies with larger cohorts and improved tissue quality are warranted to clarify the clinical and biological significance of these findings further.

## 5. Conclusions

In summary, our findings indicate that TERT promoter mutations are infrequent in ameloblastoma. Due to the low mutation rate and limited sample size, definitive conclusions could not be drawn. Further studies with larger cohorts are needed to elucidate the molecular landscape of ameloblastoma and determine whether TERT promoter mutations may serve as meaningful biologic markers of this entity.

## Figures and Tables

**Table 1:** Clinical characteristics according to recurrence status (*n* = 46).

Variable	Category	Recur (+)	Recur (−)	*p*-Value
**Sex**, *n* (%)	Male	11 (61)	7 (39)	**0.03**
	Female	7 (25)	21 (75)	
**Age**, years	Range (median)	10–79 (46.5)	8–77 (23)	0.2
**Location**, *n* (%)	Mandible	13 (33)	26 (67)	0.09
	Maxilla	5 (71)	2 (29)	
**Tumor size**, cm	Range (median)	1.0–7.0 (2.5)	0.9–9.6 (3.5)	0.3

Values are presented as the number (%) or range (median); *p*-values were calculated using Fisher’s exact test for categorical variables and the Wilcoxon rank-sum test for continuous variables.

**Table 2:** Summary of TERT-Promoter-Mutation-Positive Cases and Recurrence Patterns.

Case	Recurrence Pattern	Episode	TERT Status	Tissue Availability/ Analysis
1	Recurrent	Primary	Wild type	Available/Successful
		1st recurrence	Wild type	Available/Successful
		2nd recurrence	C228T + C250T	Available/Successful
2	Non-recurrent	Primary	C250T	Available/Successful

## Data Availability

The original contributions presented in this study are included in the article. Further inquiries can be directed to the corresponding author.
